# Theoretical System of Contact-Mode Triboelectric Nanogenerators for High Energy Conversion Efficiency

**DOI:** 10.1186/s11671-018-2764-2

**Published:** 2018-10-30

**Authors:** Huamin Chen, Yun Xu, Jiushuang Zhang, Weitong Wu, Guofeng Song

**Affiliations:** 1 Institution of Semiconductors, Chinese Academy of Sciences, Beijing, 100083 China; 2 College of Materials Science and Opto-Electronic Technology, University of Chinese Academy of Sciences, Beijing, 100049 China

**Keywords:** High efficiency, Energy conversion, Force, Triboelectric nanogenerators, Flexible electronics

## Abstract

As the rapid expansion of next-generation electronics, portable and efficient energy sources has become one of the most important factors impeding the market development. Triboelectric nanogenerators (TENGs) are a potential candidate for its unsurpassed features. Herein, we deeply analyzed the power and conversion efficiency of contact-mode TENGs considering the whole energy conversion process. Firstly, reaching beyond the conventional analysis, a compressive force was introduced to derive a more versatile motion profile, which provided a better understanding of the working principle of contact-separation process. Then, we deeply analyzed the influence of various parameters on its performance. Especially, the maximum efficiency TENGs can be obtained under optimum force. It is realistic and useful for more efficient TENGs. Furthermore, this research stands a good chance of establishing standards for quantifying the efficiency of TENGs, which lays the basis for the further industrialization and multi-functionization of TENGs technology.

**Electronic supplementary material:**

The online version of this article (10.1186/s11671-018-2764-2) contains supplementary material, which is available to authorized users.

## Background

The artificial intelligence and cloud network are gradually improving the quality of our modern life with the rapid development of next-generation electronics for smart home, health monitoring, entertainment, and environment monitoring [1–3]. Powering these large amounts of electronics has become an impossible mission utilizing existing battery technologies considering its large size, short lifetime, and especially fast-charging problems. It has become one of the most important barriers to develop sustainable power source suitable for wearable electronics [4–6].

Currently, triboelectric nanogenerators (TENGs) based on the triboelectrification have been demonstrated an attractive technology for harvesting mechanical energy. It is a promising candidate for wearable electronics for its numerous advantages, including flexibility [7], cost-effectiveness [8], simple fabrication process [9], environmental protection [10], and versatility [11]. It has been widely utilized to harvest energy from ambient mechanical energy. Furthermore, it can be utilized to integrate with wearable device for self-powered applications [12–14]. For now, many methods have been utilized for increasing the power, including surface morphology [15, 16], material optimization [17, 18], charge injection [19, 20], structure optimization [21, 22], and multi-nanogenerators [23, 24]. Despite the rapid advancement in output performance, a definitive model for analyzing the energy conversion efficiency is absent. A number of theoretical explanations have been published for different modes of TENGs [25–27]. However, most analysis does not discuss the whole energy conversion process and only focus on the output power. More importantly, higher output power does not mean higher energy conversion efficiency and may even prove counter-productive. It has somewhat impeded the development of more efficient TENGs on account of lack of direct study on energy conversion efficiency.

In this work, we systemically and directly analyzed the power and conversion efficiency of contact-mode TENGs considering the whole process. Firstly, reaching beyond the conventional analysis, a compressive force was introduced to derive a more versatile motion profile, which provided a better understanding of the working principle of contact-separation process. Then, according to the motion equations, explicit equations for the important device performance in the whole contact and separation process were presented. Finally, the influence of material properties, structural parameters, and experimental factor on the maximum power and especially energy conversion efficiency was systematically researched. We can obtain the maximum efficiency and power by rationally designing parameters especially compressive force. It is realistic and useful for more efficient TENGs. Importantly, it stands a good chance of establishing the standards for quantifying the efficiency of TENGs, which lays the basis for the further industrialization and multi-functionization of TENGs.

## Methods

**Fig. 1:**
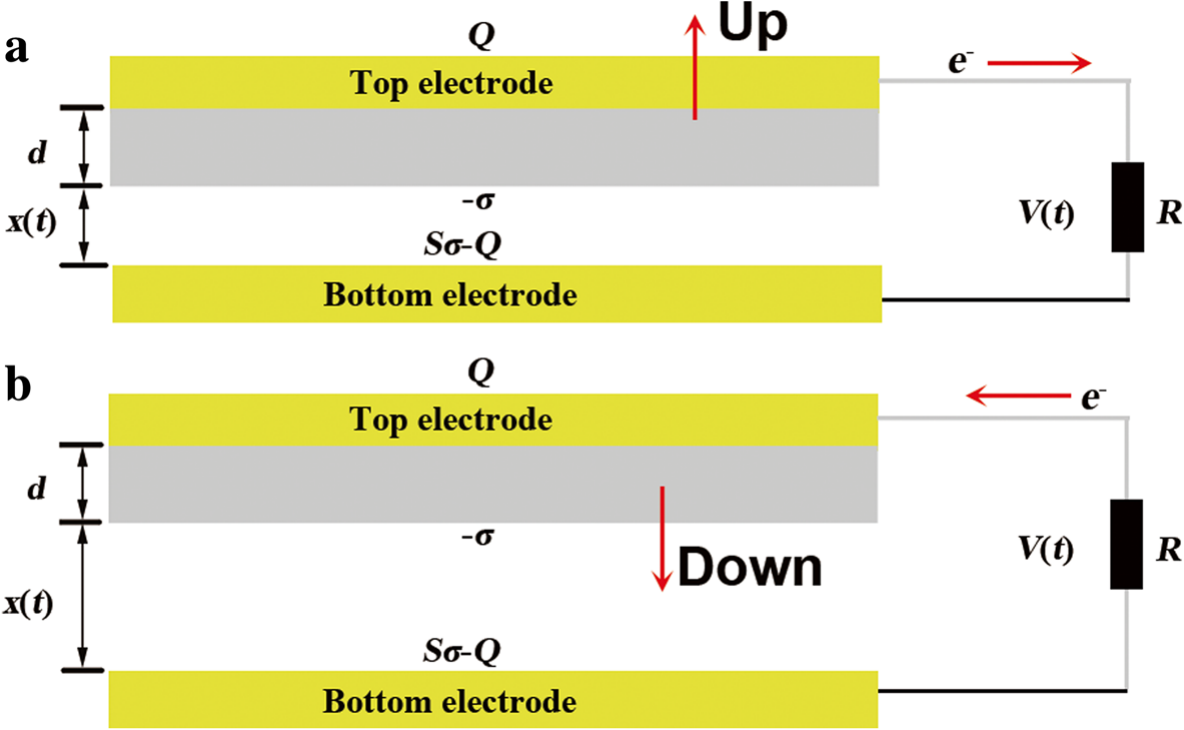
The theoretical model for contact-mode of TENGAs. **a** Separation process and **b** contact process

The basic operating principle of TENGs is built upon triboelectrification and electrostatic induction. It could be approximately classified into two types in view of the friction materials. Due to the work function and friction, the dielectric material and conductor material are chosen as the triboelectric pairs. As displayed in Fig. 1, the upper layer consist of top electrode (TE) and dielectric layer can move up and down, whereas the bottom electrode (BE) is fixed on the substrate. The two layers are connected with a load resistor *R*. The separation and contact process are indicated in Fig. 1a, b, respectively. In the separation process, electrons flow to the BE from the TE, and return to the TE in the contact process.

Under the applied force *F*, the upper layer will make full contact with the bottom layer. The BE will have positive triboelectric charges with the surface charge density *σ* while the dielectric layer has the same charges with opposite sign. In the separation process, the upper layer separates with the bottom layer with a distance *x*(*t*). It will result in a potential difference *V*(*t*) between the TE and BE due to the electric field. To offset *V*(*t*), electrons will flow between the two electrodes through *R*. Therefore, the charges of the TE is *Q* while the BE is left with *Sσ* − *Q*. The electric field strength at the two regions is given as follows according to the Gauss theorem.

Inside the dielectric layer:

1 $$ {E}_{\mathrm{dielectric}}=-\frac{Q}{S{\varepsilon}_0{\varepsilon}_r} $$

Inside the air gap:

2 $$ {E}_{\mathrm{air}}=\frac{\sigma_0-Q/S}{\varepsilon_0} $$

where *ε*_0_ and *ε*_*r*_ are the vacuum permittivity and the relative permittivity, respectively.

The *V*(*t*) should satisfy the following equation:

3 $$ V(t)={E}_{\mathrm{dielectric}}d+{E}_{\mathrm{air}}x(t) $$

From the Ohm’s law, the *V*(*t*) is given as

4 $$ V(t)= RI(t)=R\frac{dQ}{dt} $$

Merging equations, we can get

5 $$ \frac{dQ}{dt}+\frac{d_0+x(t)}{RS{\varepsilon}_0}\times Q=\frac{\sigma x(t)}{R{\varepsilon}_0} $$

The Eq. (5) is the governing equation of TENGs. It can be applied to the whole separation and contact process. It is obvious that *x*(*t*) is one of the most important factors of TENGs. Different from previous work, we build the practical motion equation rather than assuming it directly. In this paper, the motion equation in the whole process is built based on the compressive force and experimental condition.

## Results and Discussion

### Non-Spring System

Firstly, we only consider a constant compressive force *F* and the gravity of the top layer. The motion equation can be obtained as follows (see Additional file 1: Note 1 and Figure S1 in the ESM). In reality, the *x*(*t*) always has a maximum value *x*_max_ and minimum zero. So the motion equations is given by

6.1 $$ \left\{\begin{array}{c}\ x(t)=\frac{F- mg}{2m}{t}^2,t<\sqrt{\frac{2{x}_{\mathrm{max}}m}{F- mg}}\ \\ {}x(t)={x}_{\mathrm{max}},t\ge \sqrt{\frac{2{x}_{\mathrm{max}}m}{F- mg}}\end{array}\right. $$

6.2 $$ \left\{\begin{array}{c}\ x(t)=\frac{F+ mg}{2m}{t}^2,t<\sqrt{\frac{2{x}_{\mathrm{max}}m}{F+ mg}}\ \\ {}x(t)=0,t\ge \sqrt{\frac{2{x}_{\mathrm{max}}m}{F+ mg}}\end{array}\right. $$

The Eqs. (6.1) and (6.2) represent the separation process and contact process, respectively.

Then we can get the transferred charge. (The detailed derivation is in Additional file 1: Note 2 in the ESM).

In the separation process:

7.1 $$ {\displaystyle \begin{array}{l}Q(t)=\exp \left(-\frac{6m{d}_0t+\left(F- mg\right){t}^3}{6 mRS{\varepsilon}_0}\right)\\ {}\times {\int}_0^t\frac{\sigma \left(F- mg\right){t}^2}{2 mR{\varepsilon}_0}\mathit{\exp}\frac{6m{d}_0t+\left(F- mg\right){t}^3}{6 mRS{\varepsilon}_0} dt,t<\sqrt{\frac{2{x}_{\mathrm{max}}m}{F- mg}}\end{array}} $$

7.2 $$ {\displaystyle \begin{array}{l}Q(t)=\frac{\sigma S{x}_{\mathrm{max}}}{d_0+{x}_{\mathrm{max}}}-\left(\frac{\sigma S{x}_{\mathrm{max}}}{d_0+{x}_{\mathrm{max}}}-{Q}_0\right)\\ {}\times \mathit{\exp}\left(-\frac{d_0+{x}_{\mathrm{max}}}{RS{\varepsilon}_0}\left(t-{t}_0\right)\right),t\ge \sqrt{\frac{2{x}_{\mathrm{max}}m}{F- mg}}\end{array}} $$

where ${t}_0=\sqrt{2{x}_{\mathrm{max}}m/\left(F- mg\right)}$, and *Q*_0_ = *Q*(*t*_0_) in Eq. (7.1).

In the contact process:

8.1 $$ {\displaystyle \begin{array}{l}Q(t)=\exp \left(-\frac{6m{d}_0t+\left(F+ mg\right){t}^3}{6 mRS{\varepsilon}_0}\right)\\ {}\times \left(\sigma S+{\int}_0^t\frac{\sigma \left(F+ mg\right){t}^2}{2 mR{\varepsilon}_0}\mathit{\exp}\frac{6m{d}_0t+\left(F+ mg\right){t}^3}{6 mRS{\varepsilon}_0} dt\right),t<\sqrt{\frac{2{x}_{\mathrm{max}}m}{F+ mg}}\end{array}} $$

8.2 $$ Q(t)={Q}_0\times \exp \left(\frac{d_0{t}_0-{d}_0t}{RS{\varepsilon}_0}\right),t\ge \sqrt{\frac{2{x}_{\mathrm{max}}m}{F+ mg}} $$

where ${t}_0=\sqrt{2{x}_{\mathrm{max}}m/\left(F+ mg\right)}$, *Q*_0_ can be calculated by assigning *t* = *t*_0_ into Eq. (8.1).

Therefore, the output current can be derived as *I*(*t*) = *dQ*/*dt* and *V*(*t*) = *RI*(*t*).

According to the specific parameters exhibited in Table 1, we can obtain the numerical calculation results.

**Table 1:** The specific parameters used in the model

Symbol	Value
Surface charge density σ	40 μC/m^2^
Relative permittivity *ε*_*r*_	2.8
Thickness of dielectric layer *d*	1 × 10^−4^ m
Effective thickness of dielectric layer *d*_0_ = *d*/*ε*_*r*_	3.57 × 10^−5^ m
Area *S*	2 × 10^−3^ m^2^
Maximum distance *x*_max_	2 × 10^−3^ m
Mass of the upper layer *m*	20 g
Total spring coefficient *k*	8 N/mm
Compressive force *F*	20 N

**Fig. 2:**
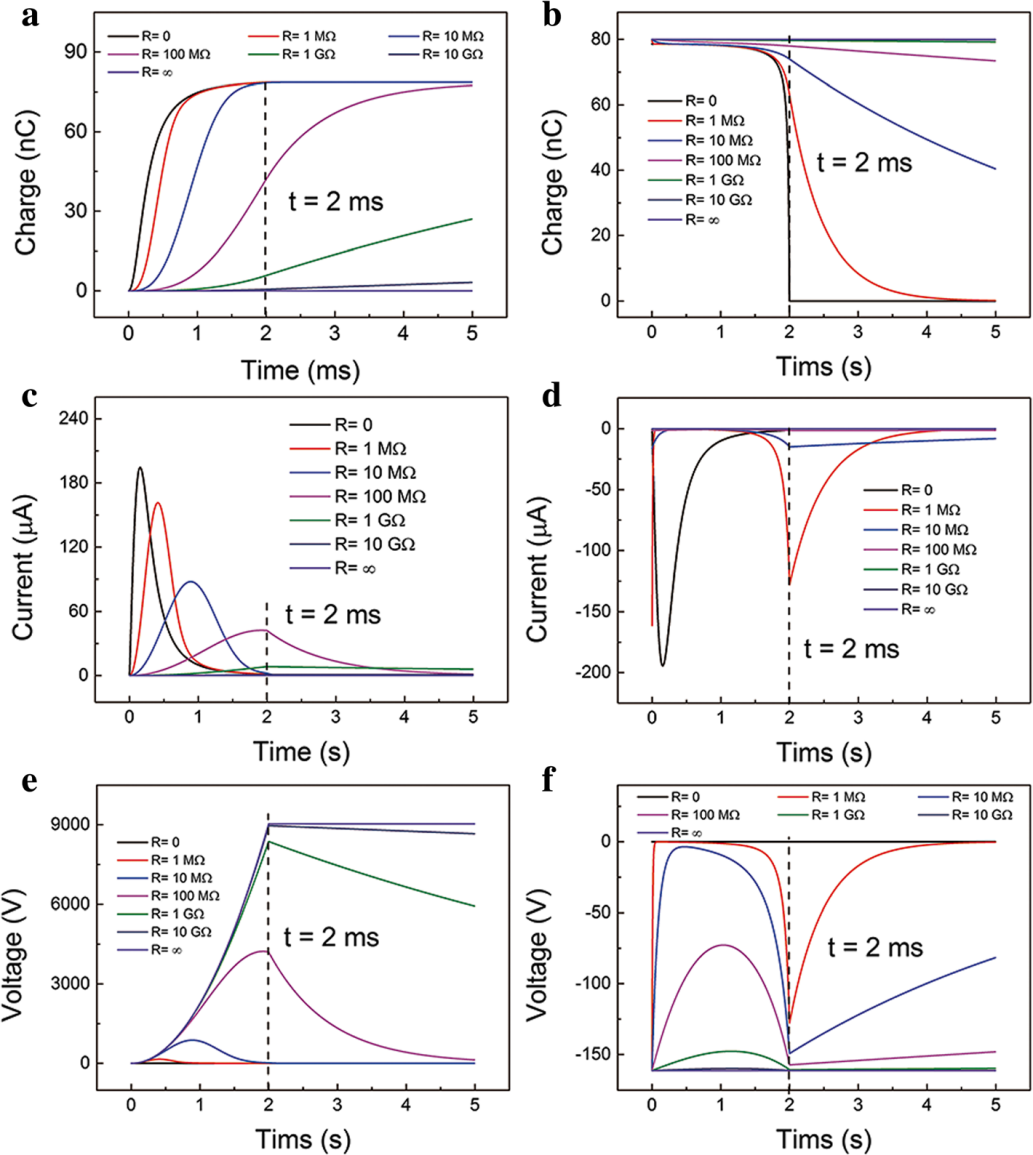
Calculated output characteristics when the device is under a constant compressive force *F.* Transferred charges-time relationship at different *R* in the **a** contact process and **b** separation process. Current-time relationship at different *R* in the **c** contact process and **d** separation process. Voltage-time relationship at different *R* in the **e** contact process and **f** separation process

The characteristics-time relationship at different *R* in the whole process is displayed in Fig. 2. The transferred charges, output current, and output voltage relationships at different loads in the contact process are shown in Fig. 2a, c, e. The behaviors are similar to the previous studies [25]. But the separation process is rarely studied. Assume that the surface charges are fully transferred to the TE in the separation process after a long time. As shown in Fig. 2b, at short-circuit (SC) condition, the charges in the TE can fully flow back to the BE when the upper layer stops moving (*t* = 2 ms). The charges cannot decrease to zero at *t* = 2 ms when *R* is more than 1 MΩ. Whereas, nearly all the charges are transferred to the BE when *R* is less than 10 MΩ in the separation process. The transferred charges in the contact process are much less than the separation process. This is contributed to the relatively small driving force in the early contact process. The output current-time relationship is plotted in Fig. 2d. At SC condition, the peak current is nearly the same as in the separation process. When *R* is larger, the current-time curve has two local maximum value, which is at the beginning and the end of the movement. And the absolute maximum current drops dramatically as the resistance increasing. The two local maximum values at the beginning and the end of the movement are due to the adequate electrons and high-speed motion, respectively. The output voltage has the same profile with the current, but a different trend in magnitude, as shown in Fig. 2f (see Additional file 1: Figure S2 for the detailed relationship in the ESM). It should be noted the absolute maximum voltage value is much smaller compared with that in the separation process. Obviously, the voltage and current are not symmetric in the separation and contact process. Combining the separation and contact process, the output voltage and current are alternating.

**Fig. 3:**
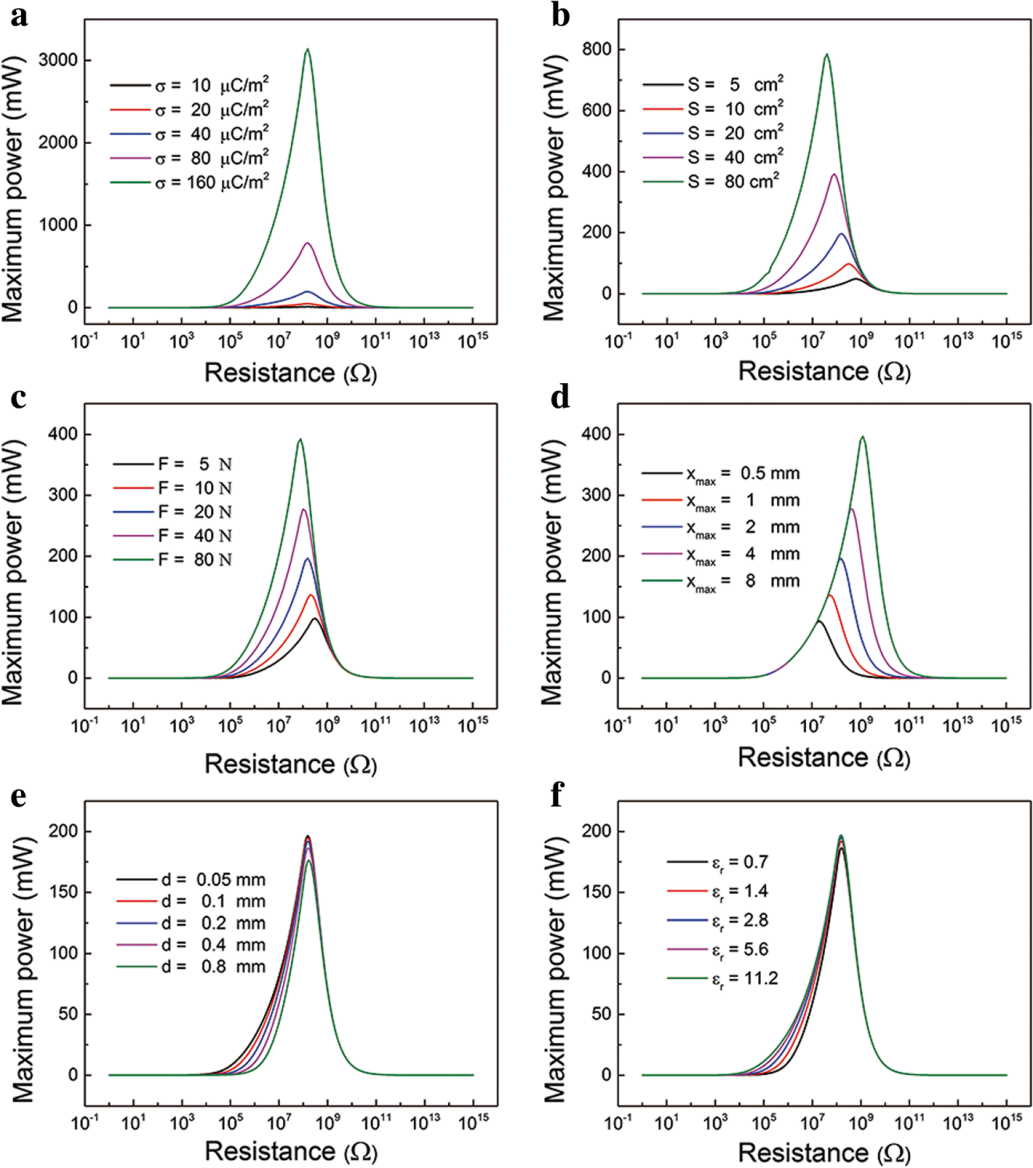
The effect of parameters on *P*_max_ and corresponding resistance. Instantaneous power profile with *R* at different **a** surface charge density σ, **b** area size *S*, **c** compressive force *F*, **d** maximum separation distance *x*_max_, **e** thickness of the dielectric layer *d*, and **f** *ε*_*r*_

In addition, the influence of various parameters on the relationship between maximum power *P*_max_ and corresponding resistance are plotted in Fig. 3. These different parameters can be divided into material, structure, and experiment condition. For example, the material parameters include the σ and *ε*_*r*_. The structural parameters are mainly area size *S, x*_max_ and *d.* The compressive force *F* is experiment parameter. One can see that σ, *S*, *F*, and *x*_max_ greatly influence the *P*_max_, as shown in Fig. 3a–d. The *P*_max_ increases dramatically as σ, *S*, *F*, and *x*_max_ increase. The parameter σ and *S* mainly decide the amount of charges that can be transferred. The parameters *F* and *x*_max_ mainly influence the motion equations. The corresponding optimum resistance decreases with *x*_max_ decreasing while it is rarely affected by σ, *S*, and *F.* In addition, the parameters *d* and *ε*_*r*_ rarely affect the *P*_max_ and corresponding resistance, as indicated in Fig. 3e, f. That is the effective thickness of dielectric layer *d*_0_ = *d*/*ε*_*r*_which has little influence on the performance of TENGs. We can adjust these parameters to control the maximum power. It is worth noting that corresponding resistance is usually the load resistance of electronics.

### Spring System

**Fig. 4:**
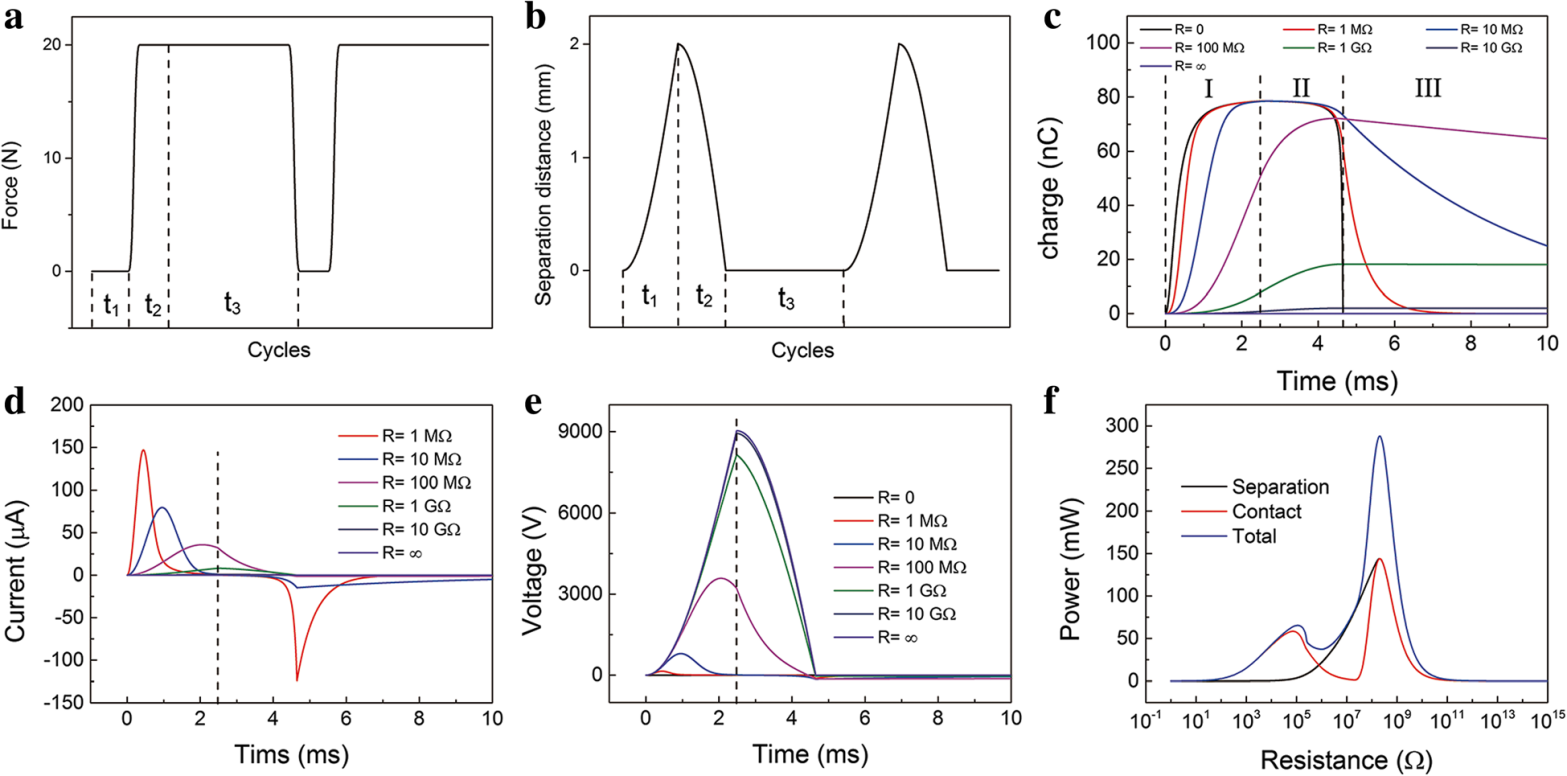
Calculated characteristics of the contact-separation mode TENGs*.* **a** The periodic force *F*. **b** The periodic motion of the top layer. **c** Transferred charges-time relationship at different *R* in the contact and separation process. **d** Current-time relationship at different *R* in the contact and separation process. **e** Voltage-time relationship at different *R* in the contact and separation process. **f** The relationships of instantaneous maximum power with resistances in the contact, separation, and the whole process

For a more popular experiment condition, the spring system is included. The compressive force *F* is applied periodically, as exhibited in Fig. 4a. In the separation process (*T* = *t*_1_), there is only restoring force of springs and gravity, so *F* = 0. While in the contact process (*T* = *t*_2_ + *t*_3_), the compressive force *F* is added. And it would still last long after the two layers into full contact. The motion curves are shown in Fig. 4b. The calculated motion equations and output performance are derived as follows. (Additional file 1: Note 3 in the ESM)

9.1 $$ \mathrm{x}(t)={x}_{\mathrm{max}}-{x}_{\mathrm{max}}\mathit{\cos}\left({\omega}_0t\right) $$

9.2 $$ \mathrm{x}(t)={x}_{\mathrm{max}}-\frac{F}{k}+\frac{F}{k}\cos \left({\omega}_0t\right) $$

where ${\omega}_0^2=k/m$. And the Eqs. (9.1) and (9.2) represent the separation process and contact process, respectively.

In the separation process:

10 $$ {\displaystyle \begin{array}{c}Q(t)={\int}_0^t\frac{\sigma {x}_{\mathrm{max}}\left(1-\mathit{\cos}\left({\omega}_0t\right)\right)}{R{\varepsilon}_0}\mathit{\exp}\left(\frac{d_0+{x}_{\mathrm{max}}}{RS{\varepsilon}_0}t-\frac{x_{\mathrm{max}}}{RS{\varepsilon}_0{\omega}_0}\mathit{\sin}\left({\omega}_0t\right)\right) dt\\ {}\times \mathit{\exp}\left(-\frac{d_0+{x}_{\mathrm{max}}}{RS{\varepsilon}_0}t+\frac{x_{\mathrm{max}}}{RS{\varepsilon}_0{\omega}_0}\mathit{\sin}\left({\omega}_0t\right)\right),t<{t}_1\end{array}} $$

In the contact process:

11 $$ {\displaystyle \begin{array}{l}Q(t)=\mathit{\exp}\left(-\frac{d_0+{x}_{\mathrm{max}}-\frac{F}{k}}{RS{\varepsilon}_0}t+\frac{Fsin\left({\omega}_0t\right)}{kRS{\varepsilon}_0{\omega}_0}\right)\\ {}\times \left\{{q}_0+{\int}_0^t\mathit{\exp}\left(\frac{d_0+{x}_{\mathrm{max}}-\frac{F}{k}}{RS{\varepsilon}_0}t-\frac{Fsin\left({\omega}_0t\right)}{kRS{\varepsilon}_0{\omega}_0}\right)\right.\ \\ {}\ \\ {}\times \left.\frac{\sigma \left({x}_{\mathrm{max}}-\frac{F}{k}+\frac{F}{k}\cos \left({\omega}_0t\right)\right)}{R{\varepsilon}_0} dt\right\},t<{t}_2\ \end{array}} $$

12 $$ Q(t)={Q}_0\times \mathit{\exp}\left(\frac{d_0}{RS{\varepsilon}_0}\left({t}_0-t\right)\right),t\ge {t}_3 $$

where *q*_0_ is the charges that transferred from the BE to the TE in the separation process.

The output current and voltage can be calculated as *I*(*t*) = *dQ*/*dt* and *V*(*t*) = *RI*(*t*).

The transferred charges-time relationship at different *R* in the full process is plotted in Fig. 4c. The charge transferred process is divided into three regions, which is corresponding to the periodic force. Region I represents the separation process and the contact process contains the regions II and III. In region I, the charges are transferred to the TE from the BE. The charges in the TE continue to increase. In region II, the direction of charge flow is related to the resistance. The charges in the TE continue to increase when the resistance is large (*R* ≥ 1 GΩ). It increases to the maximum then decrease when the resistance is low (*R* ≤ 100 MΩ). Especially, when *R* = 0, the charges will continue to decrease in region II. In region III, the charges in the TE continue to decrease. The corresponding output current in the whole process is shown in Fig. 4d. The current in the separation and contact process has the opposite sign. Usually, the maximum current value in the separation process is a bit bigger than that in the contact process. Interestingly, in the contact process, the absolute maximum current value appears at the beginning of the contact process or the moment they are just in contact. When the resistance is large, it appears at the beginning of the contact process, vice versa. The output voltage increases with time then decreases, as shown in Fig. 4e. The output voltage would appear as a negative value in the contact process. And the absolute value is much smaller than that in the separation process. These figures are consistent with measured experimental graphs in the literatures. The measured output current is alternating obviously, and the measured output voltage is usually peak-sharp. The relationships of instantaneous maximum power with resistances in the contact, separation, and the whole process are shown in Fig. 4f. The TENGs reaches its absolute maximum instantaneous power around 200 MΩ in the separation and contact process. While in the contact process, it has an additional local maximum value around 0.1 MΩ. So in the whole process, the instantaneous power gets its maximum value around 200 MΩ. One can see that the power curve of the contact process overlaps on that of separation process when the resistance is large. Because the maximum current value appears at the intersection of the two process when *R* ≥ 200 MΩ.

**Fig. 5:**
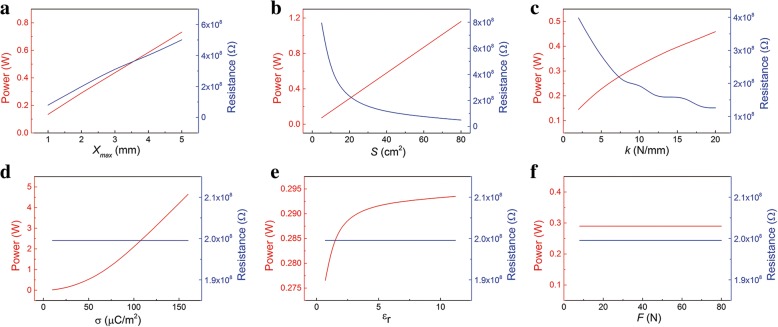
Influence of the parameters on the *P*_max_ and corresponding resistance in one cycle. The relationship of *P*_max_ and corresponding resistance with the parameters **a** *x*_max_, **b** *S*, **c** *k*, **d** σ, **e** *ε*_*r*_, and **f** *F*

Moreover, the calculated results of *P*_max_ and corresponding optimum resistance are plotted in Fig. 5. As indicated in Fig. 5a–c, the maximum instantaneous power increases as the parameters *x*_max_, *S*, and *k* increase. This can be contributed to the faster transfer speed of the electrons. At the same time, the corresponding optimum resistance also changes. The optimum resistance decreases with *S* and *k* increasing, but the reverse trend with *x*_max_. The influence of parameters σ on the *P*_max_ and optimum resistance is shown in Fig. 5d. The *P*_max_ increases rapidly with the σ increasing, while optimum resistance keeps constant. The optimum resistance is also not affected by *ε*_*r*_. But as *ε*_*r*_ increases, the maximum instantaneous power increases and then gets saturated. The *F* has nearly no influence on the maximum instantaneous power and optimum resistance. In the whole contact and separation process, the *F* only affects the contact process. So the maximum current in the separation process keeps the same. As illustrated in Fig. 5f, the maximum instantaneous power does not change. This is different from the non-spring system. In the non-spring system, the *F* directly affects the separation process, thus affects the maximum power.

In a word, the *P*_max_ can be increased by increasing the maximum separation distance *x*_max_, area *S*, spring coefficient *k*, relative permittivity of dielectric layer *ε*_*r*_, and especially surface charge density σ. For example, material parameters such as *ε*_*r*_ and σ are usually optimized to get higher power [28, 29]. While the optimum resistance can be adjusted by the parameters *x*_max_, *S*, and *k*. The *P*_max_ and optimum resistance mainly depend on the material and structural parameters.

### Conversion Efficiency *η* of TENGs

Sometimes we are more concerned about *P*_max_ that we ignore the *η*. Efficiency is an important parameter to evaluate a power source. *η* is defined as the ratio between the output electric energy and the input mechanical energy. Here, we have systematically and directly researched the influence of these parameters on the efficiency.

The electric energy and mechanical energy are obtained according to the current pulse at optimum *R*. The output electric energy is given by

13 $$ {E}_{\mathrm{electric}}={\int}_{t_{\mathrm{start}}}^{t_{\mathrm{end}}}{I}^2 Rdt $$

where the time span between *t*_start_ and *t*_end_ expresses a whole contact and separation process.

The calculated mechanical energy is

14 $$ {E}_{\mathrm{mechanical}}=F\times S $$

Thus the *η* is calculated as follows

15 $$ \eta =\frac{E_{\mathrm{electric}}}{E_{\mathrm{mechanical}}}\times 100\% $$

**Fig. 6:**
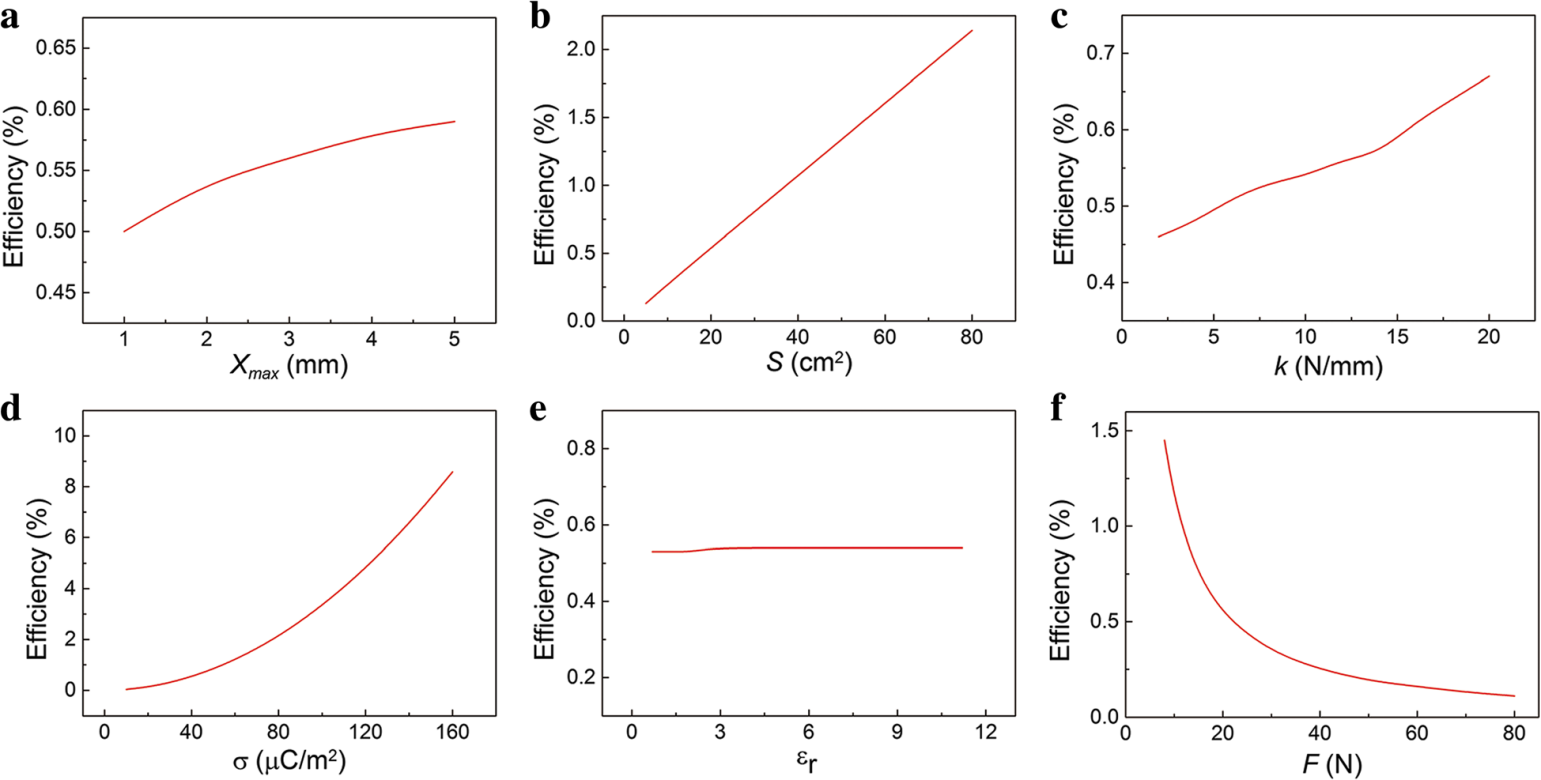
Conversion efficiency *η* of TENG. The relationship of the calculated conversion efficiency with the parameters **a** *x*_max_, **b** *S*, **c** *k*, **d** σ, **e** *ε*_*r*_, and **f** *F*

The relationship of *η* with *x*_max_ was shown in Fig. 6a. As *x*_max_ increases, the efficiency *η* increases and then get saturated gradually. We know that the mechanical energy and maximum power is proportional to *x*_max_. However, increasing *x*_max_ will change the sharp of the current-time curve. It means growth rate will slow down when *x*_max_ is larger. The influence of parameters *S*, *k*, and σ on *η* are shown in Fig. 6b–d. The increasing trend of the efficiency *η* with these parameters is similar to that of the maximum power. The efficiency *η* gradually increases with the *S* and *k* increasing. Remarkably, the σ can greatly influence the efficiency *η*. The parameter *ε*_*r*_ is difficult to change, and fortunately, it nearly not affect *η* as shown in Fig. 6e. As shown in Fig. 6f, the efficiency *η* decreases rapidly as *F* increases. This is mainly contributed to the increasing of mechanical energy. Obviously, the efficiency is relatively low. Fortunately, we can greatly increase the efficiency by improving σ.

**Fig. 7:**
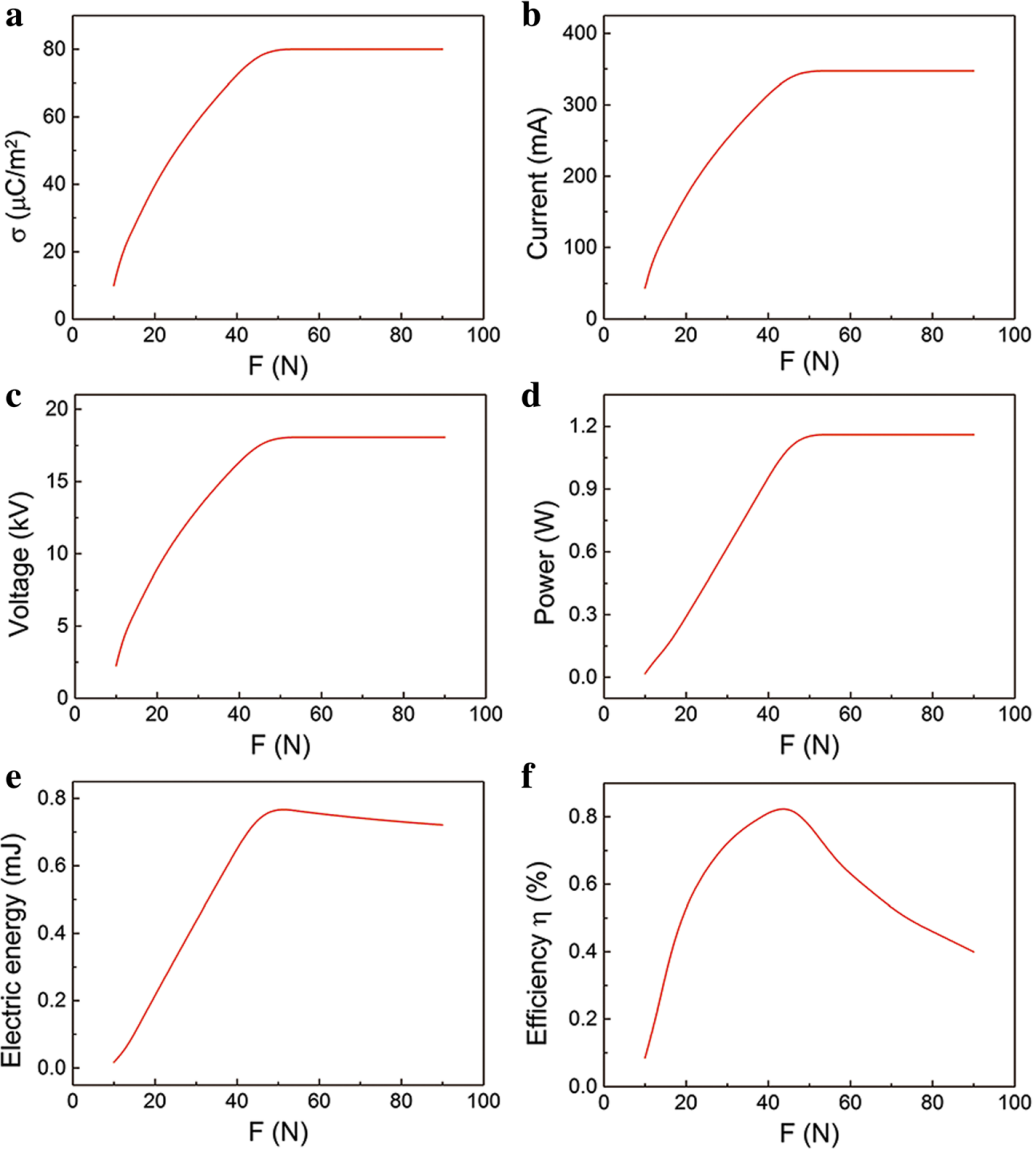
The relationship of output performance with the compressive force *F* in practical situation. **a** The influence of compressive force *F* on **a** surface charge density σ, **b** maximum current, **c** maximum voltage, **d** maximum instantaneous power, **e** electric energy, and **f** efficiency

In practical situation, however, the *F* can affect the parameter σ [30]. Under small *F*, the two layers are partial contact. The two layers can get better contact as *F* increases. Then the parameter *F* can nearly affect the surface charge density σ. That is the σ increases with the *F* then gets saturated, as shown in Fig. 7a. Therefore, we recalculated the relationship between the output performance and the compressive force *F*. The influence of *F* on maximum current, voltage, and instantaneous power are shown in Fig. 7b–d, respectively. They have the similar relationship with *F*. For example, the output voltage increases with *F* increasing and then keep constant, which is consistent with the experiment data in the literature [31, 32]. The influence of *F* on electric energy is shown in Fig. 7e. It should be noted that there is a turning point in the curve. The output electric energy increases with *F* increasing and then decreases slightly. The slight drop of output electric energy is due to the shorter contact process under larger *F*. Under small compressive force, the *F* is proportional to the σ, which results to larger output electric energy. However, under a large compressive force, the σ gets saturated. The transferred charges in the separation process keep constant while decrease in the contact process under larger compressive force. So the output electric energy in the whole separation and contact process drops slightly. The relationship of *η* and *F* is presented in Fig. 7f. Interestingly, the *η*-*F* curve is a peak-sharp and the maximum value appeared at *F* ≈ 50 N. The input *E*_mechanical_ is proportional to *F*, while *E*_mechanical_ is much larger than output *E*_electric_. Under small *F*, the growth rate of *E*_electric_ is faster than *E*_mechanical_ due to the rapid increase in σ. However, under large *F*, the decrease of *E*_electric_ and increase of *E*_mechanical_ result in a lower efficiency. The turning point in the relationship between energy conversion efficiency and compressive force is important in the design of effective power source.

In order to get higher output performance like current and instantaneous power, a large compressive force *F* is usually applied. But that may cause a low conversion efficiency. According to the above analysis, we can choose a rational *F* to get the high power as well as the conversion efficiency.

## Conclusion

In conclusion, we introduced a practical approach to systemically and directly analyze the conversion efficiency of contact-mode TENGs. Reaching beyond the conventional analysis, a compressive force was introduced to derive a more versatile motion profile, which provided a better understanding of the operating principle of contact-separation process. The explicit equations for the important device performance in the whole separation and contact process were presented, unlike the conventional analysis that only focus on the separation process. Firstly, we analyzed the relationship of output performance with material, structure, and experimental parameters, which was mainly for higher output power. Then, importantly, we systemically and deeply studied the influence of these parameters on energy conversion efficiency in the whole process. Importantly, a turning point was found in the relationship between conversion efficiency and compressive force. The TENGs with high output power and high conversion efficiency can be obtained at the same time under optimum force. It is realistic and useful for more efficient TENGs. Importantly, it stands a good chance of establishing the standards for quantifying the efficiency of TENGs, which lays the basis for the further industrialization and multi-functionization of TENGs.

## Additional file

Additional file 1: **Note 1.** Detailed calculation of the motion equations. **Figure S1.** Motion curves of the top plate in the (a) separation process and (b) contact process. **Note 2.** Detailed calculation of the output performance. **Figure S2.** (a) The relationship of maximum output voltage and current with the load resistance. (b) The relationship of instantaneous power with the load resistance. **Note 3.** Detailed calculation of the motion equations and output performance including the spring system. (DOCX 3925 kb)

## Abbreviations

BE: Bottom electrode
TE: Top electrode
TENGs: Triboelectric nanogenerators
