# Supplementary material for: Theoretical System of Contact-Mode Triboelectric Nanogenerators for High Energy Conversion Efficiency
Source: Nanoscale Res Lett. 2018 Oct 30;13:346. doi: 10.1186/s11671-018-2764-2 (PMC6207610; doi:10.1186/s11671-018-2764-2)
Supplement: Supplementary file 1 — Note 1. Detailed calculation of the motion equations. Figure S1. Motion curves of the top plate in the (a) separation process and (b) contact process. Note 2. Detailed calculation of the output performance. Figure S2. (a) The relationship of maximum output voltage and current with the load resistance. (b) The relationship of instantaneous power with the load resistance. Note 3. Detailed calculation of the motion equations and output performance including the spring system. (DOCX 3925 kb) [file 11671_2018_2764_MOESM1_ESM.docx]

Electronic Supplementary Material

**Theoretical system of contact-mode triboelectric nanogenerators for high energy conversion efficiency**

Huamin Chen^1,2^, Yun Xu^1,2,^ ^*^, Jiushuang Zhang^1,2^, Weitong Wu^1,2^, Guofeng Song ^1,2^

^1^Institution of Semiconductors, Chinese Academy of Sciences, Beijing 100083, China

^2^College of Materials Science and Opto-Electronic Technology, University of Chinese Academy of Sciences, Beijing 100049, China

*Corresponding author.

E-mail addresses: [xuyun@semi.ac.cn](mailto:xuyun@semi.ac.cn)

**Note 1: Detailed calculation of the motion equations**

In the separation process, the top plate is affected by gravity and a constant tensile force. It is a uniformly accelerated motion.

|  | $F-mg=ma$ | (S1) |
| --- | --- | --- |

where *F* is the tensile force applied on the top plate, *m* is the mass of the top plate, and *g* is gravity, respectively.

In the experimental cases, the movement of the top plate always has a maximum distance $x_{max}$. As $x\left( t \right)$ reaches $x_{max}$, the top plate stops moving.

So the motion equations of the top plate can be expressed as:

|  | $\left\{ \begin{aligned} x\left( t \right) =\frac{F-mg}{2m}t^{2}, t<\sqrt{\frac{2x_{max}m}{F-mg}} \\ x\left( t \right) = x_{max}, t\geq\sqrt{\frac{2x_{max}m}{F-mg}} \end{aligned} \right.$ | (S2) |
| --- | --- | --- |

In the contact process, it is also a uniformly accelerated motion.

|  | $F+mg=ma$ | (S3) |
| --- | --- | --- |

The motion equations can be expressed as:

|  | $\left\{ \begin{aligned} x\left( t \right) =\frac{F+mg}{2m}t^{2}, t<\sqrt{\frac{2x_{max}m}{F+mg}} \\ x\left( t \right)=0, t\geq\sqrt{\frac{2x_{max}m}{F+mg}} \end{aligned} \right.$ | (S4) |
| --- | --- | --- |

**
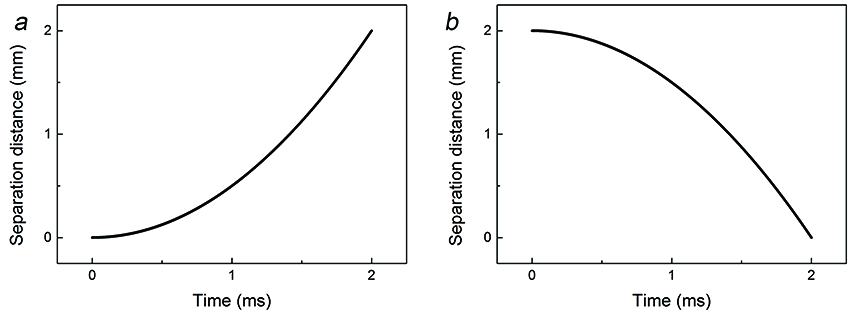
**

**Figure S1. Motion curves of the top plate in the (a) separation process and (b) contact process.**

**Note 2: Detailed calculation of the output performance.**

In the separation process, the boundary condition is

|  | $Q\left( t=0 \right)=0$ | (S5) |
| --- | --- | --- |

According to the specific boundary condition, the first-order ordinary differential equation can be solved as

|  | $\begin{matrix} Q\left( t \right)=exp\left( -\frac{6md_{0}t+\left( F-mg \right)t^{3}}{6mRS\varepsilon_{0}} \right) \\ \times\int_{0}^{t} \frac{\sigma\left( F-mg \right)t^{2}}{2mR\varepsilon_{0}}exp\frac{6md_{0}t+\left( F-mg \right)t^{3}}{6mRS\varepsilon_{0}}dt, t<\sqrt{\frac{2x_{max}m}{F-mg}} \end{matrix}$ | (S6) |
| --- | --- | --- |

When *t* exceeds $t_{0}=\sqrt{{2x_{max}m}/{(F-mg)}}$, $x=x_{max}$. The boundary condition is that

|  | $Q\left( t=\sqrt{{2x_{max}m}/{(F-mg)}} \right)=Q_{0}$ | (S7) |
| --- | --- | --- |

So the transferred charge *Q* can be calculated as:

|  | $\begin{matrix} Q\left( t \right)=\frac{\sigma Sx_{max}}{d_{0}+x_{max}}-\left( \frac{\sigma Sx_{max}}{d_{0}+x_{max}}-Q_{0} \right) \\ \times exp\left( -\frac{d_{0}+x_{max}}{RS\varepsilon_{0}}\left( t-t_{0} \right) \right), t\geq\sqrt{\frac{2x_{max}m}{F-mg}} \end{matrix}$ | (S8) |
| --- | --- | --- |

In equation S8, $Q_{0}$ can be calculated by assigning *t* equals to $\sqrt{{2x_{max}m}/{(F-mg)}}$ into equation S6.

Therefore, the output current can be derived as $I\left( t \right)={dQ}/{dt}$.

|  | $\begin{matrix} I\left( t \right)=\frac{\sigma\left( F-mg \right)t^{2}}{2mR\varepsilon_{0}}-\frac{2md_{0}+\left( F-mg \right)t^{2}}{2mRS\varepsilon_{0}}exp\left( -\frac{6md_{0}t+\left( F-mg \right)t^{3}}{6mRS\varepsilon_{0}} \right) \\ \times\int_{0}^{t} \frac{\sigma\left( F-mg \right)t^{2}}{2mR\varepsilon_{0}}exp\frac{6md_{0}t+\left( F-mg \right)t^{3}}{6mRS\varepsilon_{0}}dt, t<\sqrt{\frac{2x_{max}m}{F-mg}} \end{matrix}$ | (S9) |
| --- | --- | --- |
|  | $\begin{matrix} I\left( t \right)=\frac{d_{0}+x_{max}}{RS\varepsilon_{0}}\left( \frac{\sigma Sx_{max}}{d_{0}+x_{max}}-Q_{0} \right) \\ \times exp\left( -\frac{d_{0}+x_{max}}{RS\varepsilon_{0}}\left( t-t_{0} \right) \right) , t\geq\sqrt{\frac{2x_{max}m}{F-mg}} \end{matrix}$ | (S10) |

Then the output voltage is $V\left( t \right)=RI(t)$.

|  | $\begin{matrix} V\left( t \right)=\frac{\sigma\left( F-mg \right)t^{2}}{2m\varepsilon_{0}}-\frac{2md_{0}+\left( F-mg \right)t^{2}}{2mS\varepsilon_{0}}exp\left( -\frac{6md_{0}t+\left( F-mg \right)t^{3}}{6mRS\varepsilon_{0}} \right) \\ \times\int_{0}^{t} \frac{\sigma\left( F-mg \right)t^{2}}{2mR\varepsilon_{0}}exp\frac{6md_{0}t+\left( F-mg \right)t^{3}}{6mRS\varepsilon_{0}}dt, t<\sqrt{\frac{2x_{max}m}{F-mg}} \end{matrix}$ | (S11) |
| --- | --- | --- |
|  | $\begin{matrix} V\left( t \right)=\frac{d_{0}+x_{max}}{S\varepsilon_{0}}\left( \frac{\sigma Sx_{max}}{d_{0}+x_{max}}-Q_{0} \right) \\ \times exp\left( -\frac{d_{0}+x_{max}}{RS\varepsilon_{0}}\left( t-t_{0} \right) \right) , t\geq\sqrt{\frac{2x_{max}m}{F-mg}} \end{matrix}$ | (S12) |

In the contact process, the boundary condition is $Q\left( t=0 \right)=\sigma S$.Similar to the above, the transferred charges can be calculated as:

|  | $\begin{matrix} Q\left( t \right)=exp\left( -\frac{6md_{0}t+\left( F+mg \right)t^{3}}{6mRS\varepsilon_{0}} \right) \\ \times(\sigma S+\int_{0}^{t} \frac{\sigma\left( F+mg \right)t^{2}}{2mR\varepsilon_{0}}exp\frac{6md_{0}t+\left( F+mg \right)t^{3}}{6mRS\varepsilon_{0}}dt) , t\geq\sqrt{\frac{2x_{max}m}{F-mg}} \end{matrix}$ | (S13) |
| --- | --- | --- |
|  | $Q\left( t \right)=Q_{0}\times\exp\left( \frac{d_{0}t_{0}-d_{0}t}{RS\varepsilon_{0}} \right), t\geq\sqrt{\frac{2x_{max}m}{F+mg}}$ | (S14) |

where $t_{0}=\sqrt{{2x_{max}m}/{(F+mg)}}$, $Q_{0}$ can be calculated by assigning ${t=t}_{0}$ into equation S13.

The output current and voltage can be calculated as $I\left( t \right)={dQ}/{dt}$ and $V\left( t \right)=RI(t)$.

**
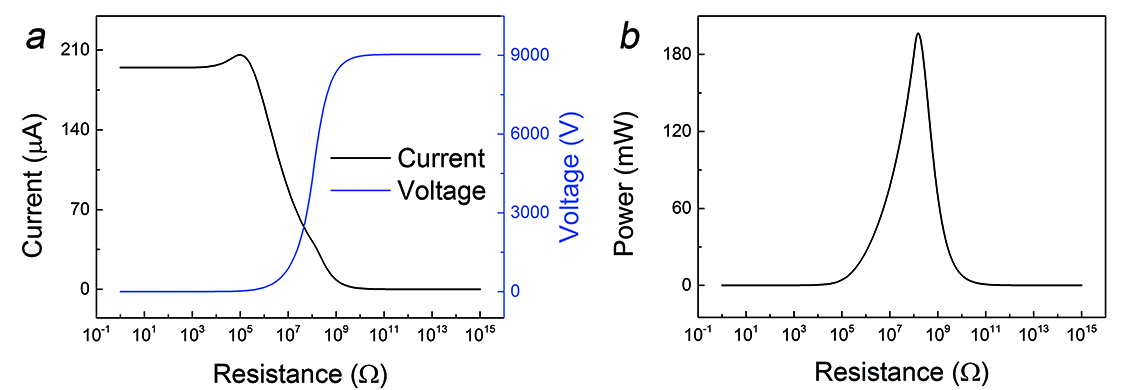
**

**Figure S2. (a) The relationship of maximum output voltage and current with the load resistance. (b) The relationship of instantaneous power with the load resistance.**

**Note 3: Detailed calculation of the motion equations and output performance including the spring system.**

In the separation process, the top plate is affected by gravity and the restoring force.

|  | $\frac{d^{2}x}{dt^{2}}+\frac{\gamma}{m}\frac{dx}{dt}+\frac{k}{m}x=0$ | (S15) |
| --- | --- | --- |

The general solution of the equation is:

$$x=Aexp\left( -\beta t \right)cos\left( \sqrt{\omega_{0}^{2}-\beta^{2}}t+\varphi\right)$$

where $2\beta=\frac{\gamma}{m}, \omega_{0}^{2}=\frac{k}{m}$ and *k* = 8 N/mm.

The boundary condition is

|  | $x\left( t=0 \right)=x_{max}$ | (S16) |
| --- | --- | --- |

Considering that the damping coefficient is very small that can be negligible, the equation can be simplified as follows:

|  | $x\left( t \right)=x_{max}cos\left( \omega_{0}t \right)$ | (S17) |
| --- | --- | --- |

The bottom plate reference point is defined coordinate origin, so the motion equation is as follows.

|  | $x\left( t \right)=x_{max}-x_{max}cos\left( \omega_{0}t \right)$ | (S18) |
| --- | --- | --- |

In the contact process, the top plate is affected by gravity, restoring force and a constant compressive force.

|  | $\frac{d^{2}x}{dt^{2}}+\frac{k}{m}x=\frac{F}{m}$ | (S19) |
| --- | --- | --- |

The general solution is

|  | $x\left( t \right)=C_{1}\exp\left( \lambda_{1}x \right)+C_{2}\exp\left( \lambda_{2}x \right)+\frac{F}{k}$ | (S20) |
| --- | --- | --- |

Considering the boundary condition $x\left( t=0 \right)=0$ and $v\left( t=0 \right)=0$, the equation is obtained as follows.

|  | $x\left( t \right)=-\frac{F}{k}\cos\left( \omega_{0}t \right)+\frac{F}{k}$ | (S21) |
| --- | --- | --- |

The bottom plate reference point is defined coordinate origin, so the motion equation is as follows.

|  | $x\left( t \right)=x_{max}-\frac{F}{k}+\frac{F}{k}\cos\left( \omega_{0}t \right)$ | (S22) |
| --- | --- | --- |

In the separation process, the boundary condition is

|  | $Q\left( t=0 \right)=0$ | (S23) |
| --- | --- | --- |

According to the specific boundary condition, the first-order ordinary differential equation can be solved as

|  | $\begin{matrix} Q\left( t \right)=\int_{0}^{t} \frac{\sigma x_{max}\left( 1-cos\left( \omega_{0}t \right) \right)}{R\varepsilon_{0}}exp\left( \frac{d_{0}+x_{max}}{RS\varepsilon_{0}}t-\frac{x_{max}}{RS\varepsilon_{0}\omega_{0}}sin\left( \omega_{0}t \right) \right)dt \\ \times exp\left( -\frac{d_{0}+x_{max}}{RS\varepsilon_{0}}t+\frac{x_{max}}{RS\varepsilon_{0}\omega_{0}}sin\left( \omega_{0}t \right) \right), t<t_{0} \end{matrix}$ | (S24) |
| --- | --- | --- |

where $t_{0}$ can be obtained from $x\left( t \right)=x_{max}$ in equation S18.

|  | $Q\left( t \right)=\frac{\sigma Sx_{max}}{d_{0}+x_{max}}-\left( \frac{\sigma Sx_{max}}{d_{0}+x_{max}}-Q_{0} \right)exp\left( -\frac{d_{0}+x_{max}}{RS\varepsilon_{0}}\left( t-t_{0} \right) \right)$, $t\geq t_{0}$ | (S25) |
| --- | --- | --- |

The output current and voltage can be calculated as $I\left( t \right)={dQ}/{dt}$ and $V\left( t \right)=RI(t)$.

In the contact process, the boundary condition is

|  | $Q\left( t=0 \right)=q_{0}$ | (S26) |
| --- | --- | --- |

where $q_{0}$ is the charges that transferred from the bottom plate to the top plate in the separation process.

|  | $\begin{matrix} Q\left( t \right)=\left( q_{0}+\int_{0}^{t} \frac{\sigma{(x}_{max}-\frac{F}{k}+\frac{F}{k}\cos\left( \omega_{0}t \right))}{R\varepsilon_{0}}exp\left( \frac{d_{0}+x_{max}-\frac{F}{k}}{RS\varepsilon_{0}}t-\frac{F}{kRS\varepsilon_{0}\omega_{0}}sin\left( \omega_{0}t \right) \right)dt \right) \\ \times exp\left( -\frac{d_{0}+x_{max}-\frac{F}{k}}{RS\varepsilon_{0}}t+\frac{F}{kRS\varepsilon_{0}\omega_{0}}sin\left( \omega_{0}t \right) \right), t<t_{0} \end{matrix}$ | (S27) |
| --- | --- | --- |

where $t_{0}$ can be obtained from $x\left( t \right)=0$ in equation S22.

|  | $Q\left( t \right)=Q_{0}\times exp\left( \frac{d_{0}}{RS\varepsilon_{0}}\left( t_{0}-t \right) \right), t\geq t_{0}$ | (S28) |
| --- | --- | --- |

where $Q_{0}$ can be calculated by assigning ${t=t}_{0}$ into equation S27.

The output current and voltage can be calculated as $I\left( t \right)={dQ}/{dt}$ and $V\left( t \right)=RI(t)$.
